# Roles and functions of social workers in long-term care for older adults in East and North-East Asia: a mixed-methods systematic review since 2000

**DOI:** 10.3389/fpubh.2026.1772661

**Published:** 2026-02-26

**Authors:** Xin Liu, Jiayu Li, Soongyu Kim

**Affiliations:** 1 Department of Social Welfare, Jeonbuk National University, Jeonju, Republic of Korea; 2 Office of Academic Affairs, Meizhouwan Vocational Technology College, Putian, China; 3 Department of Media and Communication Studies, Jeonbuk National University, Jeonju, Republic of Korea

**Keywords:** aged, Asia, Eastern, community health services, home care services, long-term care, nursing homes, social work

## Abstract

**Introduction:**

Population ageing is accelerating in East and North-East Asia (ENEA), driving growing demand for long-term care (LTC). Social workers play a pivotal role in older adults' LTC by bridging healthcare systems and everyday life through care coordination, psychosocial support, and rights- and ethics-informed decision support. However, empirical evidence on their roles and functions in ENEA LTC remains fragmented, and region-specific synthesis is lacking.

**Methods:**

We conducted a mixed-methods systematic review of peer-reviewed English-language studies published from 2000 onward to synthesize evidence on social workers' roles and functions in older adults' LTC across institutional LTC and home- and community-based services (HCBS). Following the Preferred Reporting Items for Systematic Reviews and Meta-Analyses (PRISMA) 2020 guidelines, we included 24 studies from five ENEA jurisdictions (Japan, the Republic of Korea, mainland China, Hong Kong SAR, and Taiwan region).

**Results:**

We identified five core role domains: care coordination and case management; psychosocial assessment and support; communication facilitation, decision support, and rights/ethical advocacy; education and practice innovation; and organizational and systemic change. Social workers were particularly visible in dementia care, end-of-life care, and restraint governance, supporting service quality by strengthening continuity of care and person-centered decision-making. Role enactment was constrained by blurred mandates and accountability, uneven guidance and skills training, heavy administrative and compliance workloads, workforce instability, and weak organizational backing. Evidence linking these roles to resident- or system-level outcomes (e.g., quality of life, hospitalization, restraint use, institutionalization-related outcomes, or care-continuity indicators) was limited and heterogeneous, precluding causal inference.

**Discussion:**

Strengthening social work contributions in ENEA LTC requires clearer role mandates, setting-specific competency pathways, stronger organizational support and safety governance, improved cross-sector care-transition infrastructure, and an outcome-oriented evidence base using longitudinal, quasi-experimental, or implementation designs to test role–outcome pathways.

**Systematic review registration:**

https://www.crd.york.ac.uk/PROSPERO/view/CRD420251270020, identifier: CRD420251270020.

## 1 Introduction

### 1.1 Population aging and the LTC context in ENEA

By 2024, the Asia-Pacific region's population reached 4.8 billion, accounting for approximately 60% of the world's total population (1). The number of individuals aged 65 and above increased to 503 million (10.5% of the total population) and is projected to reach 996 million by 2050 (1). East and North-East Asia (ENEA) already has a substantially higher proportion of older adults than other sub-regions (2). As population aging accelerates, the number of older adults living with functional limitations is rising, increasing long-term care (LTC) needs.

LTC refers to a comprehensive, person-centered system of health and social support services designed to help individuals who have lost partial or full self-care abilities due to chronic illness, disability, or physical and mental functional decline. Its purpose is to maintain or restore their functional capacity, thereby ensuring their fundamental rights and human dignity (3). Against the backdrop of rapid population aging and changing family caregiving capacity, LTC reforms increasingly focus on integrated systems linking institutional LTC and home- and community-based services (HCBS). These reforms aim to strengthen continuity of care and person-centered care (PCC) through cross-sector collaboration and standardized referral pathways, supporting aging in place (4–6).

### 1.2 Background

ENEA is not a single LTC policy space. Since 2000, jurisdictions have pursued distinct LTC system-building trajectories and social work professionalization arrangements. To make this temporal–institutional heterogeneity explicit for readers, we summarize below the key LTC milestones and a parsimonious developmental staging heuristic alongside social work professionalization signals for each jurisdiction (Table 1). Japan implemented Long-Term Care Insurance (LTCI) in 2000 alongside a statutory national qualification for Certified Social Workers (7, 8), and the Republic of Korea introduced public LTCI in 2008 with statutory credentialing via examination routes (9, 10). Taiwan region launched the Ten-year LTC Plan (1.0) in 2007 and Plan 2.0 in 2017 (11, 12), whereas Hong Kong SAR developed Enhanced Home and Community Care Services (EHCCS)/Integrated Home Care Services (IHCS) (2001/2003) within a non-LTCI “services for older adults” umbrella under the Social Workers Registration Ordinance (13, 14). Mainland China initiated LTCI pilots in 2016 within broader services for older adults, while the national social work qualification examination has been in place since 2008 (15, 16). Importantly, the eligible English-language evidence base identified in this review is concentrated in five jurisdictions (Japan, the Republic of Korea, mainland China, Hong Kong SAR, and Taiwan region), and does not represent all ENEA jurisdictions.

**Table 1:** ENEA jurisdictions: LTC system trajectory and social work professionalization (since 2000).

**Jurisdiction**	**Key LTC milestone(s) since 2000**	**Broad stage (analytic label) + social work professionalization signal**
Japan	LTCI implemented (2000) (7).	Consolidated LTCI-based LTC; statutory national qualification for Certified Social Workers (8).
Republic of Korea	LTCI implemented (2008) (9).	Early → consolidating LTCI system; statutory social worker credentialing with an examination route (e.g., Grade 1) (10).
Taiwan region	LTC 10-year plan (LTC 1.0) launched (2007); LTC Plan 2.0 launched (2017) (11).	Tax-funded LTC expansion → consolidation; Social Worker Act–based licensure/examination system (12).
Hong Kong SAR	EHCCS initiated (2001); IHCS formed (2003) (13).	Non-LTCI “services for older adults” with strong HCBS/residential mix; Social Workers Registration Ordinance (Cap. 505) in place (14).
China (mainland)	LTCI pilots initiated (2016) (15).	Nascent LTC within broader services for older adults services + LTCI piloting/scale-up; national social work qualification examination launched (2008) with rapid workforce expansion (16).

Stage labels represent an analytical heuristic approach rather than a ranking or linear progress model.

Within this heterogeneous regional landscape, in comparison to a division of labor driven by medicine, social work in LTC places greater emphasis on understanding and intervening with individuals within their “social–relational–environmental” context. It integrates psychosocial care into team-based care processes through psychosocial assessment, resource linkage, case management, and interprofessional collaboration (17–19). In practice, social workers frequently confront the emotional burdens and relational tensions experienced by care recipients and their families, address access barriers in referrals and service utilization, and navigate rights-based and ethical dilemmas amid potentially competing responsibilities and interests among multiple parties (19). Consequently, social workers are key liaisons connecting the healthcare system with clients' everyday lives, particularly during care transitions between institutions, community, and home. They support these transitions through information exchange, follow-up, and active coordination, thereby strengthening continuity of care and individualized support tailored to care recipients' needs (19, 20).

Despite these theoretically and practice-relevant contributions, empirical evidence on what social workers do in ENEA LTC—and how their roles are enabled or constrained by organizational, institutional, and cultural contexts—remains fragmented. The lack of a region-specific synthesis limits the visibility of social work's core value and the identification of actionable governance and workforce levers (21–23). Moreover, evidence that directly links specific social work roles to measurable outcomes (e.g., quality of life, hospitalization, continuity indicators, restraint use, or institutionalization-related outcomes) is limited and heterogeneous. While international literature has articulated core LTC social work functions (e.g., psychosocial assessment, care coordination, and rights/ethics advocacy), it remains unclear how these functions are configured and governed within ENEA's diverse LTC system trajectories; this review is therefore needed to consolidate what is known and to identify actionable system- and workforce-level levers. Accordingly, this review synthesizes both role content and enabling conditions, to clarify what appears shared across World Health Organization (WHO)-consistent LTC contexts and what is contingent on system design, mandates, and workforce governance.

### 1.3 Purpose and review questions

Accordingly, this mixed-methods systematic review aims to delineate the core roles and functions of social workers in older adults' LTC within ENEA, examine how roles differ across service types and topic areas, and synthesize the contextual factors shaping role enactment. Specifically, this review addresses the following questions:

(RQ1) What roles and functions are reported for social workers in WHO-consistent LTC contexts for older adults in ENEA?

(RQ2) How do reported roles/functions vary across service types (institutional LTC vs. HCBS vs. mixed pathways) and key topic areas (e.g., dementia care, EOL/ACP/AD-related practices, restraint governance)?

(RQ3) What policy, organizational, and sociocultural conditions enable, constrain, or reshape role enactment, and what implications do these conditions have for continuity of care and rights-sensitive decision support?

By developing an evidence-informed “role–function” framework, this review seeks to inform workforce development, service model optimisation, and governance strategies to strengthen social work contributions to ENEA LTC.

## 2 Methods

### 2.1 Protocol and reporting

This study employed a mixed-methods systematic review (MMSR) (24) and was reported in accordance with the Preferred Reporting Items for Systematic Reviews and Meta-Analyses (PRISMA) 2020 statement (25). The protocol was registered in International Prospective Register of Systematic Reviews (PROSPERO; CRD420251270020). No amendments were made to the registered protocol.

### 2.2 Search strategy

We searched four electronic databases: web of Science, PubMed, Scopus, and ProQuest. The search was limited to peer-reviewed journal articles published in English from 1 January 2000 to 13 December 2025 (last search date). We selected 2000 as the start year because the institutionalization and expansion of LTC systems in ENEA accelerated around this period (e.g., Japan's Long-Term Care Insurance was implemented in 2000), making earlier evidence less comparable with contemporary configurations spanning institutional LTC and HCBS.

The search strategy comprised three concept blocks: (1) long-term care (“long-term care”); (2) older adults (elder^*^ OR “older adults” OR “older people” OR aging OR aging); and (3) social work (“social work” OR “social worker^*^” OR “social work practic^*^”). We used the same term set and Boolean logic across all databases; database-specific field tags and syntax were adapted, and publication type/time/language limits were applied within each platform. To maximize sensitivity, we did not add jurisdiction names to the search string; geographic eligibility (ENEA) was applied during screening according to the predefined inclusion criteria. The core Boolean string was: (“long-term care” OR “long term care”) AND (elder^*^ OR “older adult^*^” OR “older people” OR “older person^*^” OR aging OR aging) AND (“social work” OR “social worker^*^” OR “social work practic^*^”). Full, database-specific search strategies (including exact field tags, limits, and run dates) are provided in Supplementary Table S1 to enable replication.

### 2.3 Inclusion and exclusion criteria

Inclusion criteria for quantitative evidence were developed using PICOS (26), and criteria for qualitative evidence were developed using PerSPecTiF (27). These were then integrated into a unified set of eligibility criteria. Studies were included if they met all eligibility criteria (Table 2): studies were excluded if they were conducted outside ENEA, outside LTC contexts, focused on general medical social work without explicit LTC practice content, or did not report identifiable social work roles/functions relevant to LTC. Because administrative labels for services vary across ENEA jurisdictions, we operationalised “LTC contexts” using a WHO-type concept during full-text review. We did not rely solely on primary studies' self-labeling (e.g., “long-term care facility/service”). Instead, we applied a concept test anchored in the WHO definition of LTC (3), requiring that the programme/setting explicitly evidenced: (1) a target population of older adults with (or at clear risk of) functional decline; (2) ongoing, long-duration support or sustained care coordination rather than time-limited/acute episodes; and (3) substantive LTC content (e.g., sustained assistance with daily living/functional support and/or structured care coordination across providers) delivered in residential/facility care, HCBS, or hospital long-stay care (e.g., LTCH/geriatric hospitals). Broader services for older adults or older adult care services papers centered on recreation, general welfare, or acute/short-term medical social work were excluded unless an identifiable LTC pathway meeting the above concept test was described. Eligible studies were then standardized as institutional LTC, HCBS, or mixed pathways when the primary study explicitly spanned both loci or examined cross-setting transitions/integrated arrangements. For transparency, we recorded (for each included study) the verbatim local service labels/administrative framing and the WHO-type cues used for mapping, and report these in Supplementary Table S2.

**Table 2:** Eligibility criteria for study inclusion.

**Domain**	**Eligibility criteria**
Population/Participants	Older adults (typically ≥ 65 years), their family caregivers, and/or the LTC workforce.
Region	Conducted in ENEA as defined by United Nations Economic and Social Commission for Asia and the Pacific (UN ESCAP) regional classification (e.g., China; China, Hong Kong SAR; China, Macao SAR; Democratic People's Republic of Korea (DPR Korea); Japan; Mongolia; Republic of Korea.) (55).
Setting/Context	Institutional LTC and/or HCBS settings (e.g., nursing homes, residential aged care facilities, long-term care hospitals; community/home care).
Phenomenon/Focus	Social workers' roles/ functions/practice content in LTC and/or organizational/system factors shaping social work practice (mandates, governance, training, workload, support mechanisms).
Study design	Primary empirical studies using qualitative, quantitative, or mixed-methods designs.
Publication type/Language	Peer-reviewed journal articles with accessible full texts in English.

### 2.4 Study selection

**Figure 1:**
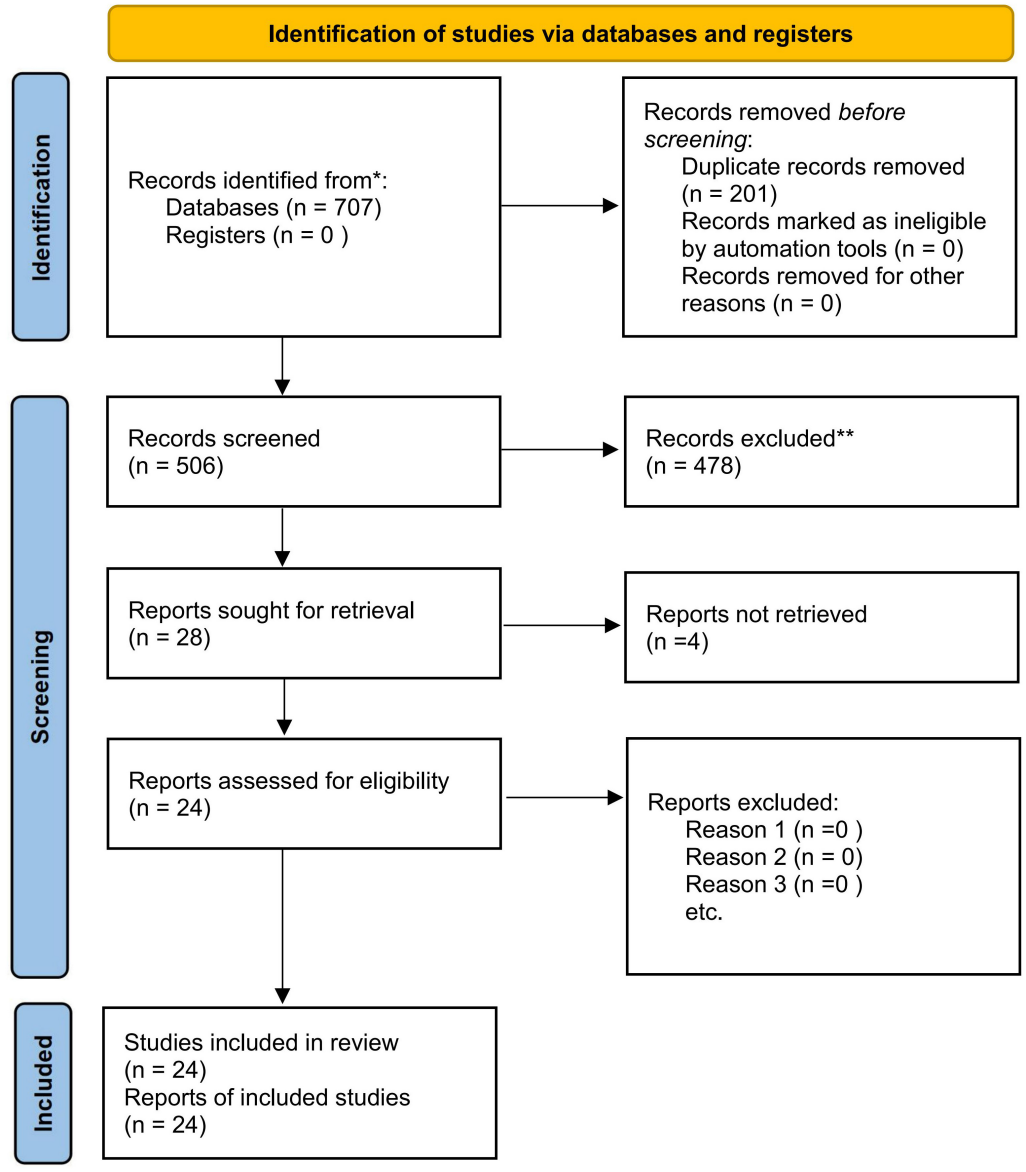
PRISMA 2020 flow diagram of study selection (25).

All records were imported into EndNote for deduplication and then uploaded to Rayyan for screening. Two reviewers (X.L. and J.L.) independently screened titles/abstracts and subsequently assessed full texts against the eligibility criteria. Disagreements were addressed through discussion; if agreement was not achieved, a third reviewer served as the arbitrator. In total, the search yielded 707 records; after deduplication, 506 unique records proceeded to title/abstract screening. Twenty-eight articles underwent full-text assessment, of which 4 were excluded due to unavailable full texts. Ultimately, 24 studies were included in the synthesis. The study-selection flow is presented in the PRISMA 2020 flowchart (see Figure 1).

### 2.5. Data extraction and quality appraisal

#### 2.5.1 Data extraction

We created and pilot-tested a standardized data-extraction template. Two reviewers (X.L. and J.L.) independently extracted and cross-checked data. Extracted items included: bibliographic information; country/area; study design and methods; participants and sample size; LTC service type/setting; topic focus (e.g., dementia, end-of-life (EOL) care, restraint governance); explicitly reported social work roles/functions and practice content; contextual and implementation conditions; and key findings and author interpretations. We did not contact study authors for missing or unclear information; no data were imputed. Primary data items were social work roles/functions and contextual/implementation conditions; when studies reported resident-, caregiver-, workforce-, or system-level outcomes (e.g., restraint use, institutionalization-related outcomes, turnover intention, continuity indicators, hospitalization, quality of life), these were extracted as secondary items as reported.

#### 2.5.2 Methodological quality appraisal

Methodological quality was appraised using the Mixed Methods Appraisal Tool (MMAT, 2018) (28). Studies were first classified by design category and then assessed against the corresponding five criteria (Yes/No/Can't tell). Two reviewers (X.L. and J.L.) conducted the appraisal independently; disagreements were resolved by discussion or third-reviewer arbitration.

### 2.6 Data synthesis

We used a convergent integrated approach (29) to integrate qualitative and quantitative evidence within a single analytical framework. To enable cross-method comparability, quantitative results were qualitized—statistical findings were translated into concise, conceptually oriented statements while retaining, where available, effect direction and key statistics (e.g., ORs, β coefficients, 95% CIs), alongside setting and participant context (30, 31). Effect estimates were extracted as reported without metric conversion or pooling, given the absence of meta-analysis. One reviewer (J.L.) performed the qualitizing, and a second reviewer (X.L.) checked the converted outputs; any discrepancies were resolved through consensus.

We then combined qualitative findings with the quantified quantitative statements and conducted thematic synthesis using a three-stage procedure: line-by-line coding, development of descriptive themes, and generation of analytical themes (32). Coding and data management were supported by ATLAS.ti (v22). An initial set of 16 descriptive themes was refined into five analytical themes (role domains). Approximately 30% of materials were double-coded, and a consensus meeting was held to finalize the thematic structure and the role–function framework.

Given substantial heterogeneity in study designs, settings, measures, and outcomes, meta-analysis was not attempted. Instead, we conducted a stratified narrative synthesis (33), organizing evidence by service type (institutional LTC vs. HCBS) and topic area (e.g., dementia, EOL care, restraint governance). Where feasible, design- and context-aware comparisons across study designs were used to assess the robustness of interpretations and to avoid causal overstatement; no formal sensitivity analyses were conducted. We did not formally assess reporting bias because no meta-analysis was conducted and outcome reporting was heterogeneous. We did not apply a certainty-of-evidence framework (e.g., GRADE) due to mixed evidence types and substantial heterogeneity; methodological quality is presented using MMAT to support cautious interpretation.

## 3 Results

Among the 24 included studies, quantitative designs predominated (*n* = 16), followed by qualitative studies (*n* = 7) and mixed-methods research (*n* = 1). Studies were conducted in Hong Kong SAR (*n* = 4), the Republic of Korea (*n* = 8), Japan (*n* = 3), mainland China (*n* = 4), and Taiwan region (*n* = 5). Service settings covered institutional long-term care (institutional LTC) only (*n* = 12; e.g., nursing homes, residential aged care facilities, and LTC hospitals), home- and community-based services (HCBS) only (*n* = 4), and cross-setting or mixed pathways (*n* = 8), including discharge transitions and integrated service arrangements. Topic foci included dementia care; end-of-life (EOL) care and advance care planning (ACP)–related practices; shared decision-making (SDM) and person-centered care (PCC) implementation; restraint governance; caregiver needs and service navigation; workforce risks and retention (e.g., client violence and turnover intentions); and inclusive practices for sexual and gender minority (SGM) older adults. Methodological characteristics and the MMAT appraisal overview are presented in Table 3. Based on the synthesis, we identified five core roles of social workers in older adults' LTC and delineated the key professional functions associated with each role (see Table 4). Standardized service-context labels (institutional LTC/HCBS/mixed pathways) were assigned by reviewers using the WHO-type concept test described in Section 2.3 rather than relying on self-labeling alone; verbatim local service terms and mapping cues are provided in Supplementary Table S2.

**Table 3:** Methodological characteristics of included primary studies (*n* = 24).

**No**.	**Study (Author, Year)**	**Country/Area^⋆^**	**Method**	**Data source/instruments**	**Participants**	***n***	**LTC service context**	**Facility type/setting details**	**Quality appraisal (MMAT)^⋆⋆⋆⋆⋆^**
1	Chan et al. (2022) (56)	HK	Mixed methods	Questionnaire; expert consensus discussion	Experts (physicians, nurses, social workers, allied health professionals)	18	Institutional LTC	Nursing home/care home	⋆⋆⋆⋆
2	Choi et al. (2023) (52)	KR	Quantitative	Survey data	Nurses; social workers; care workers	3541	Mixed pathways	LTC facilities + home care services	⋆⋆⋆⋆
3	Fukui et al. (2019) (50)	JP	Quantitative	Questionnaire; educational intervention	Home care nurses; care managers; nursing supervisors	291	HCBS	Home care/community care setting	⋆⋆⋆⋆
4	Guan et al. (40)	CN	Qualitative	Interviews	Nursing home staff (director, supervisors, nurses, care workers, social workers, interns)	17	Institutional LTC	Dementia-specific nursing home	⋆⋆⋆
5	Han (2016a) (46)	KR	Quantitative	Questionnaire; interviews	Social workers across multiple institutions	297	Institutional LTC	LTC facilities (multiple sites)	⋆⋆⋆
6	Han (2016b) (47)	KR	Quantitative	Questionnaire	Social workers in geriatric hospitals and LTC facilities	401	Institutional LTC	Geriatric hospitals; LTC facilities	⋆⋆⋆
7	Ho et al. (2016) (35)	HK	Qualitative	Focus groups	Health professionals; care home staff; family caregivers; managers	30	Institutional LTC	Care homes/ nursing homes (EOL care)	⋆⋆⋆⋆
8	Hou and Chen (2024) (57)	TW	Quantitative	Questionnaire	LTC workforce (institutional and home care staff)	542	Mixed pathways	LTC institutions + home care services	⋆⋆⋆
9	Huang et al. (2014) (49)	TW	Quantitative	Questionnaire; tests	Care staff; residents	1103	Institutional LTC	RACF(s)	⋆⋆⋆⋆
10	Huang et al. (2018) (48)	TW	Quantitative	Questionnaire	Physicians; registered nurses; social workers	478	Institutional LTC	Dementia units; nursing homes; hospital-affiliated nursing homes; general LTC facilities	⋆⋆⋆⋆
11	Huang et al. (2018) (48)	CN	Quantitative	Interviewer-administered questionnaire survey	Community-dwelling older adults	670	Institutional LTC	Integrated medical–nursing LTC facility	⋆⋆⋆
12	Kan et al. (2025) (34)	HK	Qualitative	Interviews	Service users; family caregivers; social workers; nurses; OT; PT, etc.	72	HCBS	IHCS/EHCCS programs	⋆⋆⋆
13	Kim and Kim (2023) (45)	KR	Quantitative	Questionnaire	Nurses; social workers; care workers	280	Institutional LTC	LTC facilities (SDM-focused)	⋆⋆⋆
14	Kim (2018) (42)	KR	Qualitative	Interviews	Social workers in LTC settings	15	Mixed pathways	Home care **centers**; nursing homes; day care **centers**	⋆⋆
15	Kim (2019) (43)	KR	Qualitative	Interviews	Social workers in LTC settings	15	Mixed pathways	Institutional LTC + HCBS	⋆⋆⋆
16	Kim and Yoo (2025) (53)	KR	Quantitative	Administrative database data	People with dementia	286,940	Mixed pathways	Institutional LTC + HCBS (administrative database)	⋆⋆⋆⋆
17	Ma et al. (2025) (54)	CN	Quantitative	Questionnaire	Residents in government-run rural LTC institutions (male; “Tekun” group)	718	Institutional LTC	Government-run rural LTC facilities	⋆⋆⋆
18	Mizuma et al. (2020) (37)	JP	Quantitative	Medical record review	Physicians; nurses; PT; care workers; social workers	102	Mixed pathways	LTCH discharge transition to home	⋆⋆⋆
19	Park et al. (2015) (41)	KR	Qualitative	Focus group interviews	Registered nurses; social workers	23	Institutional LTC	Nursing homes/care homes	⋆⋆⋆⋆
20	Sugisawa et al. (2025) (39)	JP	Quantitative	Questionnaire	CM	329	Mixed pathways	LTCI service system	⋆⋆⋆
21	Sun et al. (2022) (38)	CN	Quantitative	Structured interview questionnaire; needs checklist	Family caregivers of people with dementia	170	HCBS	Home-based dementia LTC	⋆⋆⋆
22	Wang et al. (2022) (36)	TW	Qualitative	Interviews	Nurses; social workers; LGBT organization staff	25	Mixed pathways	LTC facilities + community health centers	⋆⋆⋆
23	Wang et al. (2022) (44)	HK	Quantitative	DCE questionnaire (administered face-to-face)	Community-dwelling older adults using community-based LTC services	318	HCBS	Community-based LTC services	⋆⋆⋆
24	Yao et al. (2023) (51)	TW	Quantitative (quasi-experimental)	Questionnaire	Institutional residents with moderate dementia and agitation	80	Institutional LTC	LTC facility	⋆⋆

^⋆^Country/Area abbreviations: CN, mainland China; HK, Hong Kong Special Administrative Region; JP, Japan; KR, Republic of Korea; TW, Taiwan region.

Service context vs. facility type: “LTC service context” is standardized as Institutional LTC/HCBS/Mixed pathways; “Facility type/setting details” specifies the institution/service form (e.g., nursing home, RACF(s), LTCH).

ENEA, East and North-East Asia; LTC, long-term care; HCBS, home- and community-based services; LTCH, long-term care hospital(s); RACF(s), residential aged care facility/facilities; LTCI, long-term care insurance; IHCS/EHCCS, integrated EHCCS, integrated home care services/enhanced home and community care services; DCE, discrete choice experiment; SDM, shared decision-making; ACP, advance care planning; PCC, person-centered care; ADs, advance directive(s); SGM, sexual and gender minority; EOL, end of life; MMAT, Mixed Methods Appraisal Tool; OT, occupational therapist; PT, physical therapist; CM, case managers; LGBT, lesbian, gay, bisexual, and transgender.

^⋆⋆⋆⋆⋆^Quality appraisal (MMAT): The symbols indicate the MMAT star-rating shorthand: ⋆⋆ = 2-star, ⋆⋆⋆ = 3-star, and ⋆⋆⋆⋆ = 4-star. These star ratings provide a descriptive shorthand for MMAT item-level judgments; MMAT is not a numerical scoring tool.

**Table 4:** A five-category role–function framework for social workers in older adults' LTC: integrated categorization and evidence mapping.

**Roles**	**Functions**	**Supporting included studies (Table 3 study No.)**
Care coordinators and case managers	Comprehensive assessment spanning biopsychosocial and environmental domains; design and integrate care plans, then refine them iteratively as needs change; act as the primary liaison for multidisciplinary coordination; facilitate resource linkage and enhance service reach and affordability; manage transitions and support timely information exchange between agencies, community providers, and hospitals.	7, 12, 18, 23
Psychosocial assessment and support providers	Assess psychological status, family relationships, social support, and cultural context; provide emotional support, adjustment support, bereavement support, and, when needed, meaning-making and/or spiritual support; support caregivers (burden assessment, informational and emotional support, and respite resource coordination); address cultural tensions (e.g., filial piety and dependency expectations) through relational practice to mitigate stress and risks.	7, 12, 14, 15, 19, 22
Communication facilitators, decision-support providers, and rights/ethics advocates	Facilitate communication and family meetings to clarify values/preferences and support consensus-building and conflict resolution; provide education and support for ACP and advance directives (ADs), including SDM; advocate for rights (e.g., safeguarding safeguarding preferences of residents with dementia and promoting SGM inclusion/anti-discrimination); support ethical and legal compliance (informed consent, guardianship or proxy decision-making, documentation, and risk prevention), including promoting alternatives to restraints.	5, 6, 7, 9, 10, 12, 13, 14, 15, 22
Educators, innovators, and capacity builders	Provide interprofessional and ongoing education via workshops/manuals; build collaborative confidence, communication skills, and role clarity, along with EOL- and ADs-related knowledge; embed non-pharmacological interventions and programs (e.g., music therapy, resilience promotion, activity engagement) that foster participation and social interaction while also supporting restraint-alternative approaches.	3, 22, 24
Drivers of organizational and system-level change	Establish violence-prevention and incident-reporting procedures, paired with supportive supervision and psychosocial support systems; mitigate workforce risks (e.g., burnout and turnover intentions) to strengthen workforce stability; Institutionalize guidelines, standards, qualifications/ practice norms, and training systems; champion resource allocation and cross-sector collaboration to enable integrated LTC models.	2, 4, 5, 6, 9, 14, 16, 17, 22

### 3.1 Social workers as care coordinators and case managers

In ENEA research on LTC for older adults, social workers are frequently described as key actors in care integration. Their work primarily revolves around comprehensive biopsychosocial and environmental assessments, care-plan development, resource coordination, and continuity across care transitions. Studies in Hong Kong report that, in “social work-led case management,” social workers often serve as the first point of contact. They conduct comprehensive biopsychosocial and environmental assessments (which may include environmental and financial factors) and use these to integrate or iteratively adjust care plans and service packages. Simultaneously, through ongoing communication and coordination, they align care goals with service delivery by facilitating multidisciplinary collaboration among nurses, therapists, care workers, and other professionals (34). Evidence on integrated EOL care pathways highlights the need for continuity across services and clear communication among professionals. Social workers are integrated into multidisciplinary teams and participate in critical implementation phases to support transitions between care homes/residential facilities and the healthcare system, extending support to families, including bereavement care (35). In HCBS settings, social work-led case management frequently manifests as interprofessional integration and relational coordination. This includes advancing service integration through continuous follow-up, communication, and negotiation (34, 36). Notably, Wang et al. (36) provides preference-based evidence reflecting service users' preference for “regular meetings with case managers” and their perception of social workers as sources of service information/consultation partners, rather than detailing the operational processes of social work-led interprofessional integration.

This role is particularly salient during discharge planning and care transitions. Research indicates that social workers focus more intensely on social and environmental dimensions during transition assessments, identifying social and environmental factors (e.g., support networks and relationships, service-system barriers, and policy constraints). These findings inform judgments about the feasibility of returning home and the development of resource allocation plans (37). In respite care and home-based/community programs, studies report caregivers commonly facing information gaps and barriers to service access; unmet needs among family caregivers of individuals with dementia (including respite care needs) are documented through quantitative and descriptive studies (38). Furthermore, in managing care for financially challenged cases, studies indicate that care management roles (e.g., care managers/case managers) must address service utilization constraints related to affordability and optimize care arrangements through resource integration and cross-sector collaboration (39). In institutional LTC, particularly dementia care settings, research reports that factors such as staff turnover, inadequate training, and limited systemic support may undermine interprofessional collaboration, communication quality, and the sustained advancement of PCC (40, 41).

### 3.2 Social workers as psychosocial assessors and support providers

In the included studies, social workers, in their role as psychosocial assessors and support providers, primarily identify and address emotional distress, relational tensions, gaps in social support, and culturally embedded stressors experienced by older adults and their families throughout the LTC trajectory. In studies of EOL care and integrated care pathways, social workers are described as key implementers of psychosocial and adjustment support. Some studies also highlight support related to meaning-making and spirituality, with the scope extending to family members' bereavement and adaptation processes (35, 42). In studies examining “good death” experiences, social workers are described as facilitating family presence and participation through accompaniment, emotional support, and family liaison, while providing mediation and support amid complex family dynamics (42, 43). In this context, filial piety–related pressure and its associated family tensions are discussed primarily in Korean institutional EOL care studies (38).

In HCBS settings and respite services, studies more often emphasize caregiver burden and unmet needs, highlighting the importance of informational and emotional support, as well as accessible service navigation and respite resources (38). Concurrently, studies describe social workers using relational practice to address culturally shaped issues such as dependency expectations, hesitation in help-seeking, and family/caregiving tensions, sustaining engagement through trust-building and ongoing negotiation (34, 41). In addition, in research on care for SGM older adults, social workers' psychosocial support frequently co-occurs with themes of safety, dignity, and social support networks, alongside the need to respond to bias-related risks, interactional strain, and potential conflicts (44).

### 3.3 Social workers as communication facilitators, decision-support providers, and rights/ethics advocates

Across the included studies, social workers demonstrate a relatively concentrated set of functions related to communication facilitation, decision support, and rights- and ethics-related issues. Studies describe how social workers facilitate family communication and involvement (including participation in care discussions or family meetings when needed) to clarify residents' values and preferences, support conflict resolution, and incorporate family perspectives and broader social contexts into interprofessional care discussions and decision-making processes (35, 42). In social work-led interprofessional case management settings, studies also report that social workers translate service users' and families' perspectives into team decisions and action plans through information integration, ongoing negotiation, and interprofessional communication coordination (34).

In studies on SDM, social workers participate alongside nurses and care workers, and the extent of their involvement is associated with a person-centered organizational climate, communication practices, and training exposure (45). With respect to EOL care and ACP including ADs, Korean studies highlight a structural tension: although social workers are expected (or positioned) to support AD-related information exchange, discussion facilitation, and rights-related concerns within interprofessional collaboration, their roles may lack clear legal authorization, and the absence of standardized guidance may amplify ethical and legal risks and contribute to practice inconsistency (46, 47). In dementia care studies, social workers' participation, influence, and self-efficacy in EOL discussions appear more limited and are linked to continuing education and confidence, suggesting that effective involvement in these highly sensitive decisions depends on structured training and supportive collaborative mechanisms (35, 48).

In restraint governance research, social workers' involvement in consent procedures, guardianship/representation interfaces, and risk prevention is explicitly noted, and one study reports an association between social worker–signed consent and higher restraint use (49). In studies of care for SGM older adults, advocacy for non-discriminatory environments, the promotion of safety and dignity, and responses to discrimination-related risks and interactional strain are likewise presented as important components of practice (44).

### 3.4. Education and practice innovation: social workers as educators, innovators, and capacity builders

Within the ENEA LTC evidence base, social workers' educational and capacity-building functions are primarily reflected in interprofessional collaboration training, strengthening knowledge related to EOL care and ADs, and integrating non-pharmacological interventions. Studies show that structured interprofessional EOL education can improve team collaboration, confidence, and role understanding, and can support coordinated implementation of community- and home-based EOL care (50). At the same time, several studies report that social workers have comparatively limited knowledge and lower communication confidence regarding EOL discussions and decision support, and they identify continuing education as an important explanatory factor or discussion focus (46–48). In institutional LTC studies on SDM and PCC, education and training are associated with communication behaviors and person-centered practice (45).

In dementia care settings, practice innovation is often expressed through activity-based approaches and other non-pharmacological interventions. One quasi-experimental study indicates that music therapy may reduce agitation and related behavioral symptoms, offering more direct evidence for structured activity interventions that can be incorporated into institutional LTC. However, whether such interventions reduce reliance on medications or restraints is better framed as suggestive rather than definitive, and requires further evaluation using medication use and/or restraint use as outcomes (51). In addition, studies addressing self-management support and culturally shaped barriers to participation identify training needs, gaps in knowledge and capacity building, and limited managerial/organizational support as key constraints on implementation and scale-up (41, 44).

### 3.5 Social workers as drivers of organizational and system-level change

In the included studies, social workers' contributions to organizational environments, workforce stability, and institutional governance clustered into three domains: (1) risk-mitigation and staff-support mechanisms (e.g., incident reporting and supportive supervision), (2) institutional standards, qualifications, and training systems, and (3) resource allocation and broader system-level levers. A large-scale Korean study reported a significant association between client violence and turnover intention, positioning social workers—alongside other frontline staff—within organizational risk management and support agendas (52). Qualitative evidence further described social workers' emotional labor and vicarious trauma in EOL care, highlighting organizational contexts characterized by insufficient supervision and support (42). Qualitative research on PCC implementation similarly identified organizational constraints—including staff turnover, limited training, and burnout—as barriers to sustaining person-centered practice in institutional LTC (40).

At the institutional and policy level, Korean studies on ADs and EOL care described a context of limited legal mandates, lack of standardized practice guidance, and underdeveloped training systems; consequently, their results and discussions emphasized the need for standardization and workforce training (46, 47). In Taiwan, evidence on restraint governance addressed compliance and risk management through structural components such as guardianship/proxy arrangements, educational interventions, and the promotion of alternatives to restraints (49).

Regarding resource allocation and system-level effects, municipal-level evidence from South Korea reported statistical associations between community social work resources and institutionalization-related outcomes, with interpretations implicating coordination and integration with medical services (53). In comparatively resource-constrained institutional LTC settings, studies also framed staffing shortages and insufficient training as salient contextual constraints (54). Finally, in SGM older-adult care, inadequate institutionalized inclusion policies and limited managerial support were explicitly identified as key barriers, linking social worker advocacy to organizational-level change processes (44).

## 4 Discussion

This mixed-methods systematic review integrated qualitative and quantitative evidence on social workers' roles and functions in East and North-East Asia (ENEA) long-term care (LTC) and the conditions shaping role enactment. Quantitative studies identified where roles were most visible and reported associations among key variables across settings (e.g., client violence and turnover intention; community social work resources and institutionalization-related outcomes; SDM and person-centered organizational climate/training). Qualitative studies provided contextual accounts of how roles were enacted in routine practice and how organizational environments shaped judgments, risk management, and professional boundaries (35, 42, 45, 52, 53). However, evidence linking specific roles to measurable outcomes was limited and heterogeneous, precluding causal inference or pooled effect estimates. Across the included studies, outcome evidence was most frequently reported for restraint use, institutionalization-related outcomes, workforce turnover intention, and continuity/transition indicators; evidence on quality of life and hospitalization was sparse. The five role domains plausibly influence resident- and system-level outcomes through three recurring pathways: (i) continuity and coordination across transitions, (ii) person-centered communication and rights-sensitive decision support, and (iii) risk governance and workforce stability. Given the evidence base, these pathways are presented as synthesis-informed mechanisms rather than causal claims. Beyond role classification, the synthesis highlights cross-level conditions that enable or constrain these mechanisms.

### 4.1 Diversity in role structures and cross-contextual coherence along a shared throughline

The review findings point to five core social work roles in LTC: care coordination and case management; psychosocial assessment and support; communication facilitation, decision support, and rights/ethical advocacy; education and practice innovation/capacity building; and organizational and system-level change. Despite variations in welfare systems, service delivery structures, and professional divisions across ENEA regions, the included studies still suggest a common thread: social work interventions foreground the social–relational–environmental dimensions of care and support person-centered service planning and cross-service coordination, guided by rights, dignity, and care recipients' preferences. Given cross-jurisdictional variation in administrative boundaries around LTC (especially where broader services for older adults umbrellas are used), the convergence we report should be interpreted as convergence in roles/functions within WHO-consistent LTC contexts, rather than equivalence of local programme classifications or administrative service boundaries. Drawing on studies of discharge transitions, care pathway integration, and case management, social workers often act as key liaisons, translating plans and coordinating follow-up between the medical system and clients' everyday lives. Through social context assessment, resource coordination, and interprofessional communication, they translate individual needs into actionable care arrangements (34, 35, 37).

Interpreting the five roles through the developmental stages in Table 1 helps distinguish institutionalized practice from emergent or aspirational functions. In more consolidated LTCI-based systems (e.g., Japan; the Republic of Korea), care coordination/case management and interprofessional collaboration are more likely to be benefit-defined and routinised with clearer accountability and training pathways, whereas in non-LTCI “services for older adults” umbrellas (Hong Kong SAR) and mainland China's broader “yanglao fuwu” environment, similar functions are more often programme-contingent and shaped by local resourcing, mandates, and workforce availability. Accordingly, system-facing roles (e.g., safety governance, standard-setting, organizational change) tend to be more consistently evidenced where legal mandates and institutional supports are clearer, but appear partial, contested, or difficult to sustain in earlier or fragmented stages.

### 4.2 Integrating social and relational dimensions into care: key contributions of case management, care transitions, and health–social care integration

Across the included studies, social workers function as integrators within the health–social care chain, with contributions concentrated in comprehensive assessment, service coordination, resource linkage, and transition support to maintain continuity. Research on social worker-led case management in Hong Kong indicates that social workers typically serve as the primary service liaison. Through relationship coordination and persistent follow-up, they facilitated cross-disciplinary integration, transforming case management from mere “service referral” into integration work in its own right (34). In studies examining service flexibility and service-user preferences, social workers were also identified as a vital source of information. Service users expressed a preference for more frequent case management contact, reflecting a high demand for “professional information + ongoing adjustments” (36).

In transition/discharge settings, Japanese research further highlights social workers' unique assessment perspective: during discharge evaluations, social workers focus more intensely on environmental factors (social support and relationships, service-system and policy barriers, etc.), thereby providing critical evidence for determining the feasibility of returning home and resource allocation (37). Taken together, this finding indicates that as health–social care integration gains traction across the region, social workers' contributions move from a peripheral, add-on function to a more central role in coordination and continuity. Through social needs assessment, they fill gaps in medical teams' understanding of community resources, living conditions, and available support networks.

Research on respite services and dementia family care suggests that caregivers often face cost and access barriers when seeking formal support, and many report limited social support and low trust in these services. In some settings, social workers serve as key connectors, relaying information and helping people navigate access to services. This suggests that without stable resources and institutional support, care integration and continuity management are more likely to be constrained for socioeconomically disadvantaged families (38). Evidence from economically disadvantaged contexts shows that care management extends beyond health needs and is shaped by coverage rules, out-of-pocket costs, and local service availability. When institutional and cross-sectoral collaboration capacities are insufficient to support “fine-grained integration,” the effectiveness of case management becomes limited (39).

### 4.3 Preference- and rights-oriented individualized care: the role of social workers in PCC, SDM, and highly sensitive decision-making issues

Across the included studies, social workers supported shared decision-making (SDM) and person-centered care (PCC) by incorporating values, preferences, family dynamics, and social resources into care discussions through social assessment, family conferences, and communication facilitation, and by contributing to conflict mediation and implementation support (34, 35, 42). Quantitative evidence on SDM in South Korea shows that higher SDM is associated with a person-centered organizational climate, everyday communication behaviors, and exposure to PCC education. This suggests that the effective participation of social workers (and wider care teams) in decision support is highly dependent on organizational culture and capacity-building conditions, rather than solely on individual attitudes (45).

In Korean studies on end-of-life (EOL) care and advance care planning (ACP)/advance directives (ADs), institutional frictions are frequently noted: social workers often help interpret ADs documentation and facilitate conversations with patients and families, but front-line implementation remains hampered by unclear legal and procedural requirements, and shared, standardized practice guidance is still lacking. This amplifies ethical and legal risks and leads to inconsistent practice quality, highlighting the necessity for institutionalized norms and continuing education (46, 47). Across ENEA, the legal–policy infrastructure for ACP/AD implementation (e.g., statutory recognition, mandated facility procedures, and proxy/guardianship interfaces) is uneven. Where legal authorization and operational protocols are clearer, social workers' decision-support roles are more readily standardized and defensible; where mandates remain ambiguous, practice becomes more programme-contingent and risk-laden. Research on EOL discussions in dementia settings suggests that social workers often clarify advance-care documents and guide family conversations, but some report limited medical familiarity and lower confidence. This suggests that highly sensitive decision-making issues require clearer competency boundaries, training systems, and interprofessional collaboration mechanisms (48). Taiwanese evidence on physical restraints suggests that when social workers are involved in consent procedures, guardianship/proxy decision-making, and risk governance, their decision-making positions may be associated with higher-stakes or adverse outcomes. Observed differences in restraint use associated with “consent signed by social workers” indicate the need for more stringent ethical caution, robust alternatives to restraint, and educational interventions, to prevent risk governance from being reduced to procedural signing in practice (49). Finally, research on SGM older adults indicates that advocacy and cultural competence are directly linked to safety, dignity, and service accessibility; when institutionalized inclusion policies and managerial support are inadequate, related practices become more difficult to sustain consistently (44).

### 4.4 Professional practice constraints: role generalization, boundary ambiguity, and organizational attrition

While this research confirms social work's critical contributions, it also reveals several limiting factors. First, numerous studies position social workers in cross-setting supportive roles (e.g., social context assessment, emotional support, communication coordination, and service linkage), suggesting that more specialized training and institutional arrangements are needed across different care models to prevent role “generalization” and ensure the profession's irreplaceable value remains evident (34, 35). Second, multiple studies indicate that role boundaries and task divisions exhibit a blurred pattern. In areas such as communication and collaboration, plan formulation, and decision-making support, the delineation of responsibilities among social workers, nursing staff, caregivers, and case managers is often difficult to clearly define. Furthermore, unclear boundaries of authorization and competence in topics such as ACP/ADs and dementia EOL discussions may increase risks and inconsistencies (46–48). Third, the organizational environment can limit role performance in day-to-day practice. Staff turnover, inadequate training, and occupational burnout often make it difficult to put PCC and SDM into routine care. In EOL care settings, emotional labor and high-conflict situations, in the absence of supervision and support mechanisms, can substantially undermine service continuity and quality (40, 42). Furthermore, the association between client violence and turnover intentions indicates that occupational risk management and workforce stability have themselves become integral components of LTC system quality (52). Fourth, in certain high-risk scenarios (such as EOL decision-making and restraint consent), documentation, procedural accountability, and liability may constitute major sources of social workers' workload, creating tension with deeply relational practice (42, 49).

## 5 Implications and future directions

### 5.1 Evidence implications

Comprehensive evidence indicates that overcoming these limitations requires multi-stakeholder coordination within the LTC delivery system. First, governments and regulatory bodies should set out social workers' mandated roles and accountability in LTC, with explicit guidance for high-risk areas such as ACP/ADs, restraint governance, and dementia EOL communication, including clear lines of legal authority (who can decide, who can document, and who bears liability), minimum procedural safeguards, and escalation pathways for disputes or uncertainty. Establishing practical, standardized guidelines and clearly delineated authorization boundaries will reduce compliance risks and practice inconsistencies (46, 47, 49). Second, service providers should integrate organizational support into quality governance, including violence prevention and incident reporting protocols, supportive supervision, psychological support, and peer support mechanisms to mitigate emotional labor and stabilize the workforce (42, 52). Third, professional and academic organizations should offer setting-specific training curricula for HCBS and institutional LTC rather than a one-size-fits-all program. Core components may include interprofessional collaboration, PCC/SDM communication skills, EOL and ADs practice knowledge, and risk governance with ethical decision support. Intervention studies suggest that EOL education delivered across disciplines strengthens collaborative confidence and clarifies team roles. In addition, non-pharmacological interventions (e.g., music therapy) provide more actionable approaches for activity promotion, social interaction, and symptom management in institutional LTC settings (50, 51). Finally, at the system level, evidence on resource allocation indicates that greater community social work capacity may be associated with a lower likelihood of institutionalization. However, in resource-constrained regions, low social worker ratios and inadequate training may constitute structural shortcomings, further underscoring the importance of workforce development and institutionalized training systems (53, 54).

### 5.2 Future directions

Based on these findings, the East and North-East Asia (ENEA) region's long-term care (LTC) system should prioritize the following practices and policy directions:

(1) Role institutionalization and clearer lines of authority. For legally and ethically sensitive work (e.g., ACP/ADs processes, consent and guardianship interfaces, restraint governance), formalize social workers' scope of work, define accountability routes, and standardize procedures to reduce unwarranted variation and lower compliance risk.
(2) Differentiated capacity-building pathways. Establish stackable continuing-education modules and competency-based certification tracks aligned with service type (institutional LTC vs. HCBS) and thematic needs (e.g., dementia/EOL communication, SDM facilitation, ethics and legal literacy, and risk governance).
(3) Organizational support and safety governance. Put in place supportive supervision and staff-care structures, provide resources to address emotional labor and vicarious trauma, and standardize procedures for preventing, reporting, and responding to client violence to stabilize the workforce and sustain person-centered practice.
(4) Cross-sector collaboration and transition infrastructure. Operationalize social work's connector role within integrated LTC by building standardized referral pathways, shared information-exchange protocols, and formal care-transition agreements. Future studies should prioritize designs that can test specific role—outcome pathways and implementation conditions across settings.

## 6 Limitations

This review has several limitations. First, we did not search gray literature (e.g., policy documents and industry reports), which may underrepresent system-facing functions and implementation details. Second, the English-language evidence base was geographically uneven: eligible studies were concentrated in five jurisdictions (Japan, the Republic of Korea, mainland China, Hong Kong SAR, and Taiwan region), and no eligible English-language studies were identified for other ENEA jurisdictions (e.g., Mongolia, Macao SAR, DPR Korea), limiting cross-jurisdictional comparisons and the generalisability of ENEA-wide inferences. Third, heterogeneity in service contexts (institutional LTC/HCBS/mixed pathways), role labels, and outcome measures precluded meta-analysis and constrained causal interpretation; only a minority of studies reported outcomes plausibly linked to social work roles (e.g., agitation, restraint use, institutionalization-related outcomes, turnover intention, or continuity indicators). Fourth, many quantitative studies were cross-sectional or secondary analyses, limiting tests of change over time and mediating pathways, while qualitative studies were often small and setting-specific. Fifth, in some studies, social workers were not the analytic focus, reducing task-level specificity, and definitions/operationalisations of PCC/SDM and ACP/ADs varied across papers. Sixth, restricting inclusion to English-language publications may omit locally grounded evidence published in regional languages. Finally, although we applied a WHO-type concept test to operationalise LTC, variation in administrative boundaries between “LTC” and broader services for older adults across ENEA may introduce residual misclassification.

## 7 Conclusions

This mixed-methods systematic review synthesized 24 peer-reviewed English-language studies (since 2000) on social workers' roles in older adults' long-term care (LTC) in ENEA, with eligible evidence concentrated in five jurisdictions (Japan, the Republic of Korea, mainland China, Hong Kong SAR, and Taiwan region). We identified five core role domains spanning care coordination/case management, psychosocial assessment/support, communication and decision support with rights/ethics advocacy, education and practice innovation, and organizational/system-level change. Across institutional LTC, HCBS, and mixed pathways, social workers most consistently contributed by applying a social–relational–environmental lens to person-centered planning, strengthening care transitions and continuity, and supporting highly sensitive decision-making in dementia and end-of-life contexts. At the same time, role enactment was frequently constrained by blurred mandates and accountability, uneven guidance and training, heavy compliance workloads, workforce instability, and limited organizational backing. Overall, the synthesis suggests that social work's distinctive value in ENEA LTC lies in translating a social—relational—environmental assessment into coordinated, rights-sensitive care planning and transition support across institutional LTC and HCBS. Strengthening this contribution will require jurisdiction-tailored role mandates and accountability, practice-ready guidance for ACP/AD/EOL and other high-risk domains, and organizational conditions that protect time for relational work while supporting safety governance and workforce stability.

## Data Availability

The original contributions presented in the study are included in the article/Supplementary material, further inquiries can be directed to the corresponding author.
