# Supplementary material for: Roles and functions of social workers in long-term care for older adults in East and North-East Asia: a mixed-methods systematic review since 2000
Source: Front Public Health. 2026 Feb 26;14:1772661. doi: 10.3389/fpubh.2026.1772661 (PMC12980548; doi:10.3389/fpubh.2026.1772661)
Supplement: Supplementary file 1 [file Data_Sheet_1.pdf]

Supplementary Table S1. Database-specific search strategies (last run: 13 December 2025)

No.	Database	Field	Search string (as run)	Limits / filters
1	Web of Science Core Collection	TS (Topic)	*TS=("long-term care" OR "long term care") AND TS=(elder* OR "older adult*" OR "older people" OR "older person*" OR aging OR ageing) AND TS=("social work" OR "social worker*" OR "social work practic*")*	Document type: Article; Language: English; Timespan: 2000–2025
2	PubMed	[tiab] (Title/Abstract)	*("long-term care"[tiab] OR "long term care"[tiab]) AND (elder*[tiab] OR "older adult*"[tiab] OR "older people"[tiab] OR "older person*"[tiab] OR aging[tiab] OR ageing[tiab]) AND ("social work"[tiab] OR social worker*[tiab] OR social work practic*[tiab])*	Language: English; Article type: Journal Article; Publication date: From 2000/01/01 to 2025/12/13
3	Scopus	TITLE-ABS-KEY	*TITLE-ABS-KEY ("long-term care" OR "long term care") AND TITLE-ABS-KEY (elder* OR "older adult*" OR "older people" OR "older person*" OR aging OR ageing) AND TITLE-ABS-KEY ("social work" OR "social worker*" OR "social work practic*")*	Document type: Article; Language: English; Year: 2000–2025
4	ProQuest (Advanced Search)	Field = SUMMARY (All abstracts and abstract text)	*summary("long-term care" OR "long term care") AND summary(elder* OR "older adult*" OR "older people" OR "older person*" OR aging OR ageing) AND summary("social work" OR social worker* OR "social work practic*")*	Limits: Peer-reviewed Publication type: Scholarly journals Language: English Publication date: From 2000/01/01 to 2025/12/13

Note.The same conceptual term set and Boolean logic were applied across all databases. Database-specific field tags and syntax were adapted (TS in Web of Science; [tiab] in PubMed; TITLE-ABS-KEY in Scopus; Field = SUMMARY in ProQuest Advanced Search). We retained both British and American spelling variants in the database search strings to maximize retrieval. The limits/filters applied within each platform are shown above.
