# Supplementary material for: Roles and functions of social workers in long-term care for older adults in East and North-East Asia: a mixed-methods systematic review since 2000
Source: Front Public Health. 2026 Feb 26;14:1772661. doi: 10.3389/fpubh.2026.1772661 (PMC12980548; doi:10.3389/fpubh.2026.1772661)
Supplement: Supplementary file 2 [file Data_Sheet_2.pdf]

Supplementary Table S2. Study-by-study WHO-type mapping of local service labels to LTC contexts and review service categories

No.	Study (Author, Year)	Jurisdiction	Local / study-used terms (verbatim)	WHO-LTC–aligned service locus & elements evidenced	WHO LTC mapping rule (brief)	Review category (Institutional / HCBS / Mixed pathways)
1	Chan et al. (2022)	HK	care home settings / care homes	Residential care home; EOL/palliative components delivered within care home	Residential care where ongoing care/support is the primary locus → LTC	Institutional LTC
2	Choi et al. (2023)	KR	LTC facilities; Type of LTCI benefits	LTCI-defined institutional and home-care benefit settings; workforce across both	If LTCI benefit types explicitly cover home + facility →cross-setting	Mixed pathways
3	Fukui et al. (2019)	JP	HCBS; care managers; home helpers	LTCI home/community care; care management + home-help + visiting nursing; EOL-at-home collaboration	Formal LTC delivered primarily in home/community → HCBS	HCBS
4	Guan et al. (2025)	CN	nursing homes; PCDC	Nursing home dementia care; ongoing personal care + nursing services	Residential facility providing ongoing support for functional decline → LTC	Institutional LTC
5	Han (2016a)	KR	LTC facilities; EOLCD/ ADs	EOL decision-support activities embedded in LTC facilities	LTC content delivered in residential LTC facility → institutional	Institutional LTC
6	Han (2016b)	KR	geriatric hospital; LTC facility; LTC settings	Long-stay geriatric hospital + LTC facility contexts; AD/EOL knowledge and role expectations	Hospital-based long-stay care and residential LTC both fall within WHO "hospital/residential care" settings → treat as institutional for stratification	Institutional LTC
7	Ho et al. (2016)	HK	RCHE; nursing homes; palliative LTC	EOL integrated care pathway anchored in RCHE/nursing homes; hospital links are referral/transfer pathways	Primary care locus is residential LTC; hospitals as linked pathway → institutional	Institutional LTC
8	Hou & Chen (2024)	TW	LTC institutions; roles incl. home care supervisor/case manager	Workforce categories include institution-based and home-care–linked roles; study frames "LTCgivers/workforce" across LTC	Evidence indicates both institutional and home-care staffing/roles in LTC system → cross-setting	Mixed pathways

9	Huang et al. (2014)	TW	RACFs	RACFs; ongoing daily care + restraint governance/consent	Residential LTC facility context → institutional	Institutional LTC
10	Huang, H.-L. et al. (2018)	TW	LTC facility; ADs/LSTs	EOL discussion within LTC facilities (dementia units/nursing homes etc.)	Residential LTC facility delivery → institutional	Institutional LTC
11	Huang, Z. et al. (2018)	CN	institutional-based LTC; medical–social integration	Integrated medical–nursing senior care facility (institution entry as LTC receipt)	Institutional admission for ongoing care/support → institutional	Institutional LTC
12	Kan et al. (2025)	HK	HCBS; IHCS/EHCCS	Home/community care + SW-led case management; aging-in-place services	Formal LTC delivered in home/community → HCBS	HCBS
13	Kim, D.E. & Kim, M.J. (2023)	KR	LTC facilities; SDM	SDM/person-centered processes occurring within LTC facilities	Care processes embedded in residential LTC facility → institutional	Institutional LTC
14	Kim (2018)	KR	home care center; day care center; nursing home	Practice spans home care/day care + residential settings	Explicit multi-setting practice → mixed	Mixed pathways
15	Kim (2019)	KR	home care centers; adult day care; nursing homes	EOL-related SW practice across home/day + facility settings	Explicit multi-setting practice → mixed	Mixed pathways
16	Kim & Yoo (2025)	KR	home-visit services; LTC facility beds; institutional care	Administrative resources include home-visit services + facility beds	Institutional + HCBS resources jointly operationalise LTC→ mixed	Mixed pathways
17	Ma et al. (2025)	CN	government-funded welfare institutes; rural LTC facilities	Welfare institute as residence; ongoing daily support; functional assessment (ADL)	Residential long-term supportive care for functionally vulnerable older adults → institutional	Institutional LTC
18	Mizuma et al. (2020)	JP	LTCH; discharge planning to home	LTCH (hospital long-stay) + discharge planning for return home	Cross-setting transition (hospital long-stay → home) is central → mixed	Mixed pathways
19	Park et al. (2015)	KR	nursing home	Nursing home residents; chronic-disease self-management support within facility	Residential LTC facility context → institutional	Institutional LTC
20	Sugisawa et al. (2025)	JP	care managers; LTC support office	Care management under LTCI; coordinates service use under LTC system (potentially across settings)	System-level care management coordinating LTC services (not limited to one locus) → mixed	Mixed pathways

21	Sun et al. (2022)	CN	home care; adult day service; respite care	Home-dwelling PLWD; multi-domain home/community LTSS	Formal supports enabling daily functioning at home/community → HCBS	HCBS
22	Wang, Y.-C. et al. (2022)	TW	LTC facilities; community healthcare centres; home care services	Provider-facing factors shaping LTC care quality/access in facilities + community-linked contexts	LTC care delivery/access discussed across facility + community-linked settings → mixed	Mixed pathways
23	Wang, K. et al. (2022)	HK	community-based LTC service users; CCSV	Community care voucher purchasing home/community LTC services	HCBS financing/service design for community-dwelling users → HCBS	HCBS
24	Yao et al. (2023)	TW	institutional care; nursing homes	In-facility intervention for residents with dementia	Residential LTC facility context → institutional	Institutional LTC

Notes.

(1) Country/Area abbreviations: CN, mainland China; HK, Hong Kong Special Administrative Region; JP, Japan; KR, Republic of Korea; TW, Taiwan region.

(2) Review category is standardized as Institutional LTC / HCBS / Mixed pathways; "WHO-LTC–aligned service locus & elements evidenced" summarises the setting/service form (e.g., care home, RACF(s), LTCH) as described in the study and the WHO-consistent cues used for mapping.

(3) Other abbreviations: LTC, long-term care; HCBS, home- and community-based services; LTCH, long-term care hospital(s); RACF(s), residential aged care facility/facilities; LTCI, long-term care insurance; IHCS/EHCCS, Integrated Home Care Services/Enhanced Home and Community Care Services; ADs, advance directive(s); LST(s), life-sustaining treatment(s); RCHE, Residential Care Home for the Elderly; CCSV, Community Care Service Voucher; PLWD, people living with dementia; SDM, shared decision-making; PCDC, person-centred dementia care; EOLCD, end-of-life care decision-making.
